# Impact of Metabolomics Technologies on the Assessment of Peritoneal Membrane Profiles in Peritoneal Dialysis Patients: A Systematic Review

**DOI:** 10.3390/metabo12020145

**Published:** 2022-02-04

**Authors:** Antonia Kondou, Olga Begou, John Dotis, Vasiliki Karava, Eleftherios Panteris, Anna Taparkou, Helen Gika, Nikoleta Printza

**Affiliations:** 1 Pediatric Nephrology Unit, 1st Department of Pediatrics, Hippokratio Hospital, Aristotle University of Thessaloniki, 54642 Thessaloniki, Greece; antkontou@auth.gr (A.K.); yandot@auth.gr (J.D.); vasilikikarava@hotmail.fr (V.K.); 2 Laboratory of Analytical Chemistry, Department of Chemistry, Aristotle University of Thessaloniki, 54124 Thessaloniki, Greece; Olina_18@hotmail.com (O.B.); panteris@chem.auth.gr (E.P.); 3 Biomic_AUTh, Center for Interdisciplinary Research and Innovation (CIRI-AUTH), Balkan Center, B1.4, 10th km Thessaloniki-Thermi Rd, P.O. Box 8318, 57001 Thessaloniki, Greece; gkikae@auth.gr; 4 1st Department of Pediatrics, Pediatric Immunology and Rheumatology Referral Center, Hippokratio General Hospital, Aristotle University of Thessaloniki, 54642 Thessaloniki, Greece; annatapark@gmail.com; 5 Laboratory of Forensic Medicine & Toxicology, School of Medicine, Aristotle University of Thessaloniki, 54124 Thessaloniki, Greece

**Keywords:** peritoneal dialysis, peritoneal membrane, metabolomics, biomarkers

## Abstract

Peritoneal dialysis (PD) is an effective and frequent dialysis modality in adults, particularly preferred in infants and young children with end-stage renal disease (ESRD). Long-term exposure of the peritoneal membrane to dialysis solutions results in severe morphologic and functional alterations. Peritoneal dialysis effluent biomarkers are based on omics technologies, which could predict the onset or confirm the diagnosis of peritoneal membrane dysfunction, would allow the development of accurate early prognostic tools and, potentially, the identification of future therapeutic targets. The purpose of our study was to critically review the literature on the impact and the effectiveness of metabolomics technologies in peritoneal health. The main search was performed in electronic databases (PubMed/MEDLINE, Embase and Cochrane Central Register of Controlled Trials) from inception to December 2020, using various combinations of Medical Subject Headings (MeSH). The main search highlighted nine studies, of which seven were evaluated in detail. Metabolomics technologies may provide significant input in the recognition of peritoneal membrane dysfunction in PD patients and provide evidence of early intervention strategies that could protect peritoneum health and function.

## 1. Introduction

Peritoneal dialysis (PD) is a safe and common treatment modality in adults and the preferred mode of dialysis in children with end-stage kidney disease (ESKD) [1]. The method is based on the peritoneal membrane, which features as a semi-permeable barrier for ultrafiltration and diffusion [2]. Long-term exposure of the peritoneal membrane to PD solutions, multiple episodes of PD-related peritonitis and high glucose load PD solutions eventually result in peritoneal morphological and functional alterations, which may cause peritoneal membrane fibrosis, with the clinical outcome of loss of peritoneal ultrafiltration capacity [3]. Encapsulating peritoneal sclerosis (EPS) constitutes the most severe complication of peritoneal fibrosis with significant morbidity and mortality. Its incidence ranges from 2.1% to 19.4% in patients under PD of 5–8 years old and rises with PD duration [4,5]. Diagnosis is usually made in advanced stages of the disease by laparotomy or positron emission tomographic imaging [6]. The pathogenesis of peritoneal membrane dysfunction and progression to EPS remains poorly understood. No single definitive pathway has been identified and a model of multiple hits is suggested [7,8]. The mostly involved mechanisms include peritoneal inflammation and angiogenesis, as well as mesenchymal conversion of mesothelial cells, which is also called the mesothelial to mesenchymal transition (MMT) [9].

Over the last decades, researchers’ interest has been focused on identifying serum and peritoneal dialysis effluent (PDE) biomarkers related to peritoneal dysfunction in PD patients. Serum inflammatory markers, such as interleukin (IL)-6, monocyte chemoattractant protein (MCP)-1 MCP-3 and IL-8, have been implicated in the pathogenesis of chronic peritoneal inflammation process [10]. Vascular endothelial growth factor (VEGF) has been involved in the development of peritoneal angiogenesis; its serum levels increase along with PD duration, and decrease in case of glucose-free PD solutions [11]. However, the precise molecular pathways of VEGF-induced angiogenesis have not yet been fully elucidated [12]. Other factors may be also involved in the formation of new blood vessels, such as prostaglandin E2, IL-1β and tumor necrosis factor (TNF)-α [13]. Process of MMT is associated with VEGF, connective tissue growth factor and gremlin 1 in the PDE, and has been found to be correlated with the peritoneal permeability status [14,15].

Recently, the development of “omics” technologies, especially proteomics and metabolomics, combined with bioinformatics–biostatistics joint research efforts, have been shown to provide a highly promising tool in defining pathogen-specific immune fingerprints and phenotype-associated molecular signatures of PD patients. In this aim, PDE biomarkers, identified by these technologies, could predict the onset of peritoneal membrane dysfunction, may allow the early diagnosis of EPS, and may advance the development of PD fluids supplemented with specific amino acids, targeting preservation of peritoneal membrane health; however, till now, published data are limited.

The aims of our study were as follows: (1) to evaluate the predictive role of metabolomics technologies on the early recognition of peritoneal membrane dysfunction with respect to peritoneal fibrosis, MMT and EPS; (2) to investigate the impact of supplemented PD fluids on the PDE metabolic profile in PD patients.

## 2. Materials and Methods

### 2.1. General Information and Literature Search Strategy

This review conforms to the “Preferred Reporting Items for Systematic Reviews and Meta-Analyses” (PRISMA) statement [16,17] (PROSPERO 2022 CRD42022298923; available from https://www.crd.york.ac.uk/prospero/display_record.php?ID=CRD42022298923; accessed on 17 December 2021).

### 2.2. Information Sources and Search Strategy

Literature review was carried out with the aim to evaluate the impact of metabolomics technologies on the identification of early biomarkers of peritoneal membrane dysfunction in PD patients. In order to recognize metabolomics studies, computerized research was performed on the major electronic databases—PubMed/MEDLINE, Embase and Cochrane Central Register of Controlled Trials—as well as individual references for publications up to December 2020. The following search terms were used: “peritoneal dialysis” OR “peritoneal dialysis effluent” OR “peritoneal effluent” AND “metabolic” OR “metabolomics” OR “omics” AND “peritoneal fibrosis” OR “encapsulating peritoneal sclerosis” OR “epithelial to mesenchymal transition” OR “mesothelial to mesenchymal transition”.

Randomized controlled trials, observational retrospective cohorts and cross-sectional studies were considered for enrollment. Case reports, animal studies, systematic reviews, meta-analyses and editorials were excluded, as were studies published in any language other than English.

### 2.3. Study Selection

The evaluation of each study was carried out independently by the two principal investigators using a predefined form of information extraction (A.K. and O.B.). The baseline characteristics of each study considered—namely, the primary investigator, publication journal, year of publication, sample size, methodology and metabolomics-based technology—were checked twice. Any disagreement in the extracted data was resolved through discussion with a third checker (H.G.). Reference lists of all included articles were searched manually for further studies, also meeting inclusion criteria (A.K., O.B., J.D., V.K., H.G. and N.P.). Due to the limited number of identified studies and their heterogeneity, a meta-analysis was not possible.

### 2.4. Data Extraction

The primary citations obtained during the database survey were recorded in a text file according to their topics and abstracts. Variables in the database were primary study outcomes, which included: (1) recognition of early biomarkers of ΜΜΤ, peritoneal fibrosis and encapsulating sclerosis in PD patients from studies applying a metabolomics approach; (2) effectiveness of early intervention strategies to protect peritoneum health and function, based on metabolomics studies’ findings.

## 3. Results

**Figure 1:**
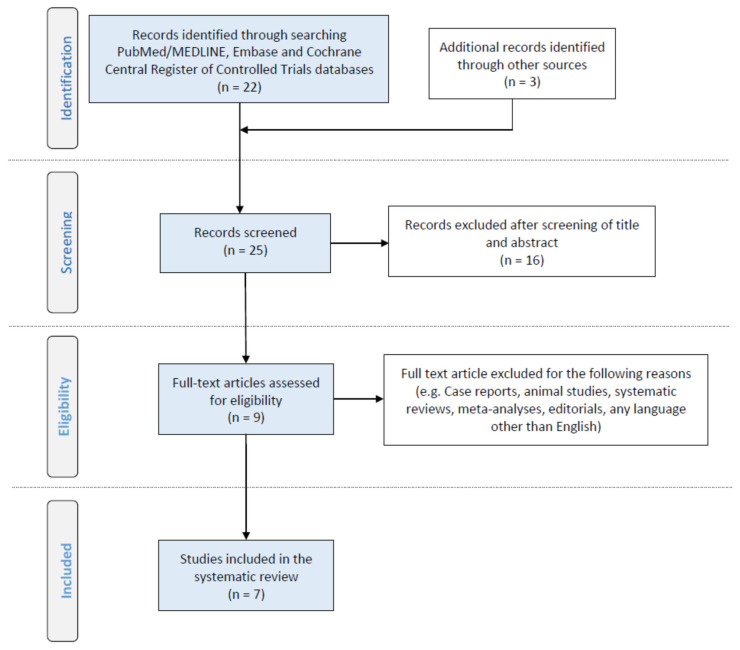
PRISMA flow diagram of literature search, eligibility and inclusion process [16,17].

The primary research highlighted nine studies, of which two were excluded as review and experimental studies, respectively (Figure 1). Seven studies were evaluated in detail and their characteristics are included in Table 1 and Table 2. It should be noted that studies showed heterogeneity regarding both the approach and the techniques used. Table 1 summarizes patient demographics, while Table 2 details the metabolomics-based techniques used, along with the results of each study.

Tang et al. [18], following an untargeted approach, investigated plasma lipid profile of patients undergoing PD therapy by a two-dimensional liquid chromatography-quadrupole time of flight (2D LC-qTOF) method, aiming to discover biomarkers that can predict PD technical failure. From 190 endogenous lipid species, and from the 10 lipid classes that were identified, 8 phospholipid species, possessing either a sphingomyelin or a phosphatidylcholine head group, significantly differed between the groups. More specifically, PS41:4, PI40:4, SM16:0, SM20:7, SM21:0, PC35:1, PC2:11 and PC42:9 counts were significantly higher in patients who experienced PD failure (*n* = 5) during a mean follow-up time of 38 months compared with PD survivors (*n* = 15), while SM21:0 was independently associated with technical failure.

The first attempt of PDE untargeted metabolic profiling took place by Dunn et al. in 2012 [19], who performed two different complementary metabolomics-based strategies, in order to investigate PDE metabolic differences in patients who developed EPS. In total, 38 metabolites presented significant changes between cases progressed to EPS during a 6-year follow-up period (*n* = 11) and matched PD survivors (*n* = 11). Dunn et al. managed to identify 27 of them, among which were the amino acids leucine-isoleucine, phenylalanine, methionine, tyrosine and β-alanine/alanine, the amines diethanolamine, ethanolamine and trimethylamine-N-oxide, and the disaccharides lactose and trehalose, as well as ascorbate-6 phosphate.

In another study, Guleria et al. [20] were able to identify 53 small endogenous compounds in untreated PDE of PD patients using ^1^H–^13^C NMR spectroscopy. PDE was found enriched in amino acids and derivatives, such as leucine, isoleucine, valine, alanine, taurine, methionine and creatinine, in organic acids, such as citric acid, fumaric acid and 2-oxoglutarate, in amines, namely trimethylamine-N-oxide and dimethylamine, in the vitamin myo-inositol, and in other small endogenous molecules. According to the authors, these metabolites may be suitable for further research regarding metabolic alterations in patients with PD-related complications.

Asano et al. [21], using an untargeted approach, tested the relationship between dialysate-to-plasma (D/P) concentration ratios of small molecules and D/P of creatinine in both serum and PD effluent, collected 4 h after peritoneal equilibrium testing (PET). In total, 574 different molecules were detected in both serum and PD effluent of 19 patients underwent PET. Their findings suggested that, along with molecular weight (MW), the charge and the protein binding rate of small molecules were related to peritoneal transport rates.

In another untargeted metabolomics study, Csaicsich et al. [22] detected 200 mases in total in PDE by a liquid chromatography–high-resolution mass spectrometry (LC-HR/AM) method; they found 29 features, mainly related to tryptophan metabolism, which presented statistically significant alteration between samples taken at time point 0 and after 4 h of PET. In an extension of this study [23] the same research group tested the possible cytoprotective effect of alanyl-glutamine (AlaGln) addition to glucose-based PD fluid. In detail, Kratochwill et al. highlighted a plethora of metabolites that significantly changed in PDE during a 4 h PET after patient treatment with standard PD solution supplemented with 8 mM of alanyl-glutamine (AlaGln). Using an LC-HR/AM method, alanyl-glutamine supplementation was found to increase leucine, isoleucine, glutamine and arginine, as well as unsaturated fatty acids and glycolipid-related metabolites, and to decrease phenylalanine, tyrosine, homocysteic acid and nucleic acids.

In the only targeted metabolomics study, Wiesenhofer et al. [24] investigated the PDE profiles of PD patients and evaluated the cytoprotective effects of AlaGln supplementation to PD fluid. In total, 184 predefined small molecules were quantified for the first time in PDE as phenylisothiocyanate derivatives. Peritonitis history, anuria or PD duration affected the concentration of several PDE metabolites, including kynurenine, tryptophan, phenylalanine, serine, valine, symmetric dimethylarginine (SDMA) and total-DMA (SDMA + Asymmetric dimethylarginine (ADMA)). Moreover, AlaGln supplementation to PD solution was found to alter oxidative stress related biomarkers. More specifically, 8 glycerophospholipids (GLPs), long-chain acylcarnitine oleoylcarnitine and methionine sulfoxide (Met-SO) were either upregulated or downregulated in the PD effluent of patients after a 4 h PET.

## 4. Discussion

The early detection of peritoneal dysfunction may allow timely prediction of PD failure and potentially permit prompt targeted therapeutic interventions in order to optimize PD survival. This is of high importance for younger pediatric patients, since PD may be the only applicable dialysis method—kidney transplantation may be not feasible, and PD is therefore required for a longer time period. The purpose of this study was to review the literature related to the role of serum and PDE metabolic profile on the early diagnosis of peritoneal membrane dysfunction in PD patients, and review the potential effects of supplemented PD fluids on PDE metabolic profile.

Metabolites are small endogenous compounds (MW < 1500 Da), considered to be the end-stage result of various metabolic processes. Therefore, metabolomic analysis of biological specimens may provide an instant “snapshot” of the metabolic phenotype in the living system under study [25,26,27,28]. Until today, no single technique is able to provide full coverage of the metabolic profile, due to the large heterogeneity and physicochemical properties of the metabolites. Various advanced technological platforms are conscripted for metabolomics-based studies, including mass spectrometry (MS), performed either by direct infusion of samples (DIMS) or by the hyphenated separation technique (e.g., gas/liquid chromatography), and nuclear magnetic resonance (NMR) spectroscopy. Both techniques allow the determination of a large number of metabolites from complex biofluids and can provide targeted and untargeted metabolic profiling [29,30]. The first evaluates a predefined panel of metabolites, usually related to the disease under study (based on the literature), or key role compounds (e.g., amino acids) involved in determined metabolic pathways. On the other hand, an untargeted approach aims for a more holistic profiling of the endogenous compounds in the specimen of interest.

The concept of employing metabolomic analysis for disease diagnosis began in the 1940s and was further developed during 1960s and 1970s [21]. However, considerable progress in metabolomics techniques has been observed during the last decade, after the pioneering work by Fiehn and Nicholson on MS and NMR, respectively [31]. In clinical studies focused on kidney function, serum metabolomic profiles may provide evidence of glomerular filtration decline in CKD patients [32]. Recently, the pertinence of metabolomic analysis on patient PDE has evocated a novel method of identifying early biomarkers of peritoneal dysfunction [33]. PD fluid is highly appropriate for metabolomics-based studies, because it is an easily available matrix that can be collected in large volumes during PD treatment; moreover, its initial metabolic composition is simple, comprising a limited number of metabolites (<20), while its composition is subtle to peritoneal membrane cell and tissue changes, due to its direct contact with the latter [34]. The clinical implications of metabolomic analysis study in PD patients is still under question [18,19,20,21,22,23,24]. Most studies have investigated the untargeted metabolic profile in serum or PDE. The principal goals of these studies were the determination of small molecule transport rate through the peritoneal membrane, the comparison of the serum or PDE metabolic profile between PD survivors and those with peritoneal membrane dysfunction, and, finally, the exploration of cytoprotective effects of supplemented PD fluid.

Concerning the clinical usefulness of metabolomics in PD, 7 studies are of interest. In the prospective cohort study realized by Dunn et al. [19], significant changes in amino acids, amines, short chain fatty acids and sugars were detected in the PDE metabolic profiles of patients, long before the diagnosis of EPS. Of interest is that the identified metabolites exhibited a similar chemical structure or function, proving the further significance of their results. Moreover, as also remarked by the authors, although EPS patients were on PD for a longer period, PD duration did not significantly differ from that of matched PD survivors. These findings limit the confounding effect of PD duration in their analysis. In the second available cohort study from Tang et al. [18], patients who progressed to PD failure had initially different plasma phospholipid profile compared with PD survivors. In particular, high plasma SM21-0 level was associated with PD failure during a 38-month follow-up period. SM is the most abundant sphingolipid in mammalian cells and increased serum levels have been associated with impaired glomerular function and albuminuria [35]. Moreover, further studies indicated that SMs may be associated with various metabolic pathways, resulting in vascular damage, increased oxidative stress and systemic inflammation [36,37]. Therefore, high plasma SM levels in PD failure patients may possibly reflect the occurrence of peritoneal vasculopathy, which usually precedes peritoneal fibrosis, resulting in inadequate solute clearance.

In 2016, Kratochwill et al. [23] performed the first-in-human trial of supplemented PD fluid. According to their findings, PD fluid supplementation with alanine-glutamine reduced cellular stress and peritoneal inflammation. Specifically, the effect of the addition of AlaGln on PD fluids significantly increased peritoneal cell expression of Hsp72, which is considered as one of the best-described human stress protecting cell proteins. Later, in 2018, Vychytil et al. [38] conducted a randomized controlled trial of supplemented PD fluid and found out that addition of alanine-glutamine to PD solutions increased the release of ΤΝF-a, CA-125 and IL-6, molecules known for their role in the process of fibrosis and inflammation. Additionally, the investigators noticed that this enrichment reduced IL-8 levels and resulted in reduced peritoneal protein loss [39]. Finally, in 2019, Wiesenhofer et al. [24] using targeted metabolic analysis confirmed the antioxidant activity of alanine-glutamine addition to PD solutions. It is worth noting that glutamine seems to be a powerful antioxidant factor, with a significant effect on the circulation of ammonia to and from tissues, which exhibits participation in various biological processes [39].

Recently, Asano et al. [21] investigated the association between peritoneal clearance of small-sized molecular uremic toxins and peritoneal creatinine clearance in PD patients. The dialysate-to-plasma concentration ratio (D/P) of some molecules was not associated with D/P creatinine. The authors underlined that other than molecular weight parameters appear to be involved in the peritoneal transport rate, such as protein charge and binding rate. Moreover, they concluded that metabolomic analysis may serve as useful method for the study of peritoneal transport status of the low molecular weight molecules, which could accumulate in PD patients.

It is obvious that PDE metabolic analysis is technically feasible and represents a reliable method for the detection of a large number of metabolites in a single measurement. Nevertheless, the heterogeneity of metabolomic techniques applied in the aforementioned studies, and the small number of recruited patients, do not allow us to draw firm conclusions regarding the usefulness of PDE metabolic analysis in clinical practice. To that end, further, larger scale cohort studies are required to evaluate the prognostic role of PDE metabolic profiles on the early detection of peritoneal membrane dysfunction; meanwhile, randomized controlled trials are necessary to assess the potential beneficial effect of supplemented PD fluids on the preservation of peritoneal membrane health.

In the future, application of metabolοmic technologies on PD patients could enhance the accurate assessment of peritoneal permeability and may clinically improve the timely identification of patients at risk for PD failure and EPS development. In addition, PDE metabolic profile study may advance our knowledge regarding the optimal composition of PD fluids targeting extension of peritoneal permeability. Finally, PDE and serum metabolic profile analysis may potentially help the clinicians towards personalized PD treatment, tailored to the needs of each patient.

## 5. Conclusions

The metabolic profile can be efficiently studied in both the serum and the PDE of PD patients, and may be useful for the assessment of peritoneal permeability and in the early recognition of peritoneal membrane dysfunction in PD patients. Moreover, metabolite-supplemented PD solutions could potentially block peritoneal inflammation and fibrosis, resulting in extension of the peritoneum membrane’s permeability. Nevertheless, clinical interpretation of the results from the few reported studies is limited due to the small number of recruited patients and the heterogeneity of applied metabolomic technologies. The clinical significance of metabolic profiling in PD patients needs further investigation.

## Figures and Tables

**Table 1:** Patients’ demographics and PD histories.

Ref/Year/Country	Type of Study	No Patients	Age (Years) Mean (Range)	PD Vintage (Years) Mean (Range)
[18]/2014/China	Prospective Cohort Duration: 38 ± 14 months	20	NA	NA
[19]/2012/England	Prospective Cohort Duration: 6 years	22	46.5 (35–59)	NA
[20]2014/India	Cross-sectional	8	ΝA	NA
[21]/2019/Japan	Cross-sectional	19	59 (46–68)	4 (2.6–5.3)
[22]/2015/Austria	Cross-sectional	8	62 (49–74)	2.5 (0.4–4.5)
[23]/2016/Austria	Cross-sectional	20	58 (47–68)	2.38 (0.8–3.46)
[24]/2018/Poland	Randomized Controlled Trial	20	58	2.4

NA—data not available.

**Table 2:** Analytical aspects of metabolomics strategies applied in the studies and findings/identified biomarkers.

Ref/Year/Country	Matrix	Compounds	Instrumentation	Sample Preparation	Biomarkers
[18]/2014/China	Plasma	190 lipid species	NPH/RPH, LC/LC-qTOF	200 μL serum + 100 μL IS extracted with 12 mL of chloroform:methanol, 2:1, *v*/*v*. Evaporation to dryness and reconstitution with 1 mL chloroform:methanol, 2:1 *v*/*v*.	PS41:4, PI40:4, SM16:0, SM20:7, SM21:0, PC35:1, PC2:11, PC42:9
[19]/2012/England	PDE	More than 100 endogenous compounds including sugars, amino acids, organic acids and others	1. GC-TOF 2. Direct infusion MS	1. 100 μL of PDE were diluted with IS and then the sample was lyophilized. 2. 100 μL of PDE were diluted with methanol for protein precipitation. After centrifugation, the clear supernatant was directly injected into the MS.	38 metabolites in total including amino acids, sugars, amines and organic acids
[20]/2014/India	PDE	53 small endogenous metabolites	^1^H-^13^C NMR spectroscopy	400 μL of untreated PDF diluted with 0.5% sodium salt of 3-trimethylsilyl-(2,2,3,3-d_4_)- propionic acid (TSP) in deuterium oxide (D_2_O).	-
[21]/2019/Japan	Serum and PDE	38 small-, middle- and large-sized molecules	CE-TOF	-	-
[22]/2015/Austria	PDE	200 features	UHPLC-ORBITRAP	Centrifugation of PDE. On-line sample clean up.	29 significant features mainly related to tryptophan metabolism
[23]/2016/Austria	PDE	200 features	UHPLC-ORBITRAP	-	leucine, isoleucine, glutamine, arginine, fatty acids, glycolipids related metabolites, phenylalanine, tyrosine, homocysteic acid, nucleic acids (AlaGln supplementation)
[24]/2018/Poland	PDE	188 endogenous compounds including amino acids, acylcarnitines, amines, glycerophospholipids, hexoses and sphingolipids	LC/FIA-QTRAP	10 μL PDE + 10 μL IS were evaporated to dryness. Derivatization with phenylisothiocyanate and evaporation to dryness. Reconstitution with 300 μL methanol + 5 mM ammonium acetate. Filtration and centrifugation.	51 metabolites, including kynurenine, tryptophan, phenylalanine, serine, valine, SDMA, total-DMA and Met-SO

CE—capillary electrophoresis; FIA—flow injection analysis; GC—gas chromatography; LC—liquid chromatography; NMR—nuclear magnetic resonance; NPH—normal phase; PDE—peritoneal dialysis effluent; RPH—reversed phase; TOF—time of flight; UHPLC—ultra-high-pressure liquid chromatography.

## Data Availability

The data presented in this study are available on request from the corresponding author. The data are not publicly available due to current project restrictions.

## Abbreviations

PDE	peritoneal dialysis effluent
PM	peritoneal membrane
NPH	normal phase
RPH	reversed phase
LC	liquid chromatography
TOF	time of flight
UHPLC	ultra-high-pressure liquid chromatography
GC	gas chromatography
MS	mass spectroscopy
NMR	nuclear magnetic resonance
CE	capillary electrophoresis
FIA	flow injection analysis

## References

[1] Gajardo M., Cano F. (2020). ABC de la diálisis peritoneal en pediatría [ABC of the peritoneal dialysis in pediatrics]. Rev. Chil. Pediatr..

[2] Devuyst O., Goffin E. (2008). Water and solute transport in peritoneal dialysis: Models and clinical applications. Nephrol. Dial. Transplant..

[3] Krediet R.T., Struijk D.G. (2013). Peritoneal changes in patients on long-term peritoneal dialysis. Nat. Rev. Nephrol..

[4] Mihalache O., Bugă C., Doran H., Catrina E., Bobircă F., Pătrașcu T. (2014). Encapsulating peritoneal sclerosis—A rare and serious complication of peritoneal dialysis: Case series. J. Med. Life..

[5] Tseng C.C., Chen J.B., Wang I.K., Liao S.C., Cheng B.C., Wu A.B., Chang Y.T., Hung S.Y., Huang C.C. (2018). Incidence and outcomes of encapsulating peritoneal sclerosis (EPS) and factors associated with severe EPS. PLoS ONE.

[6] Danford C.J., Lin S.C., Smith M.P., Wolf J.L. (2018). Encapsulating peritoneal sclerosis. World J. Gastroenterol..

[7] Nakamoto H. (2005). Encapsulating peritoneal sclerosis—A clinician’s approach to diagnosis and medical treatment. Perit. Dial. Int..

[8] Angela M., Summers A.M., Clancy M.J., Syed F., Harwood N., Brenchley P.E., Augustine T., Riad H., Hutchison A.J., Taylor P. (2005). Single-center experience of encapsulating peritoneal sclerosis in patients on peritoneal dialysis for end-stage renal failure. Kidney Int..

[9] Balzer M.S. (2020). Molecular pathways in peritoneal fibrosis. Cell Signal..

[10] Oh K.H., Jung J.Y., Yoon M.O., Song A., Lee H., Ro H., Hwang Y.-H., Kim D.K., Margetts P., Ahn C. (2010). Intra-peritoneal interleukin-6 system is a potent determinant of the baseline peritoneal solute transport inincident peritoneal dialysis patients. Nephrol. Dial. Transplant..

[11] Witowski J., Kamhieh-Milz J., Kawka E., Catar R., Jörres A. (2018). IL-17 in Peritoneal Dialysis-Associated Inflammation and Angiogenesis: Conclusions and Perspectives. Front. Physiol..

[12] Felmeden D.C., Blann A.D., Lip G.Y.H. (2003). Angiogenesis: Basic pathophysiology and implications for disease. Eur. Heart J..

[13] Kany S., Vollrath J.T., Relja B. (2019). Cytokines in Inflammatory Disease. Int. J. Mol. Sci..

[14] Ruiz-Carpio V., Sandoval P., Aguilera A., Albar-Vizcaíno P., Perez-Lozano M.L., González-Mateo G.T., Acuña-Ruiz A., García-Cantalejo J., Botías P., Bajo M.A. (2017). Genomic reprograming analysis of the Mesothelial to Mesenchymal Transition identifies biomarkers in peritoneal dialysis patients. Sci. Rep..

[15] Loureiro J., Gónzalez-Mateo G., Jimenez-Heffernan J., Selgas R., López-Cabrera M., Aguilera Peralta A. (2013). Are the mesothelial-to-mesenchymal transition, sclerotic peritonitis syndromes, and encapsulating peritoneal sclerosis part of the same process?. Int. J. Nephrol..

[16] Moher D., Liberati A., Tetzlaff J., Altman D.G., PRISMA Group (2009). Preferred reporting items for systematic reviews and meta-analyses: The PRISMA statement. J. Clin. Epidemiol..

[17] Stewart L.A., Clarke M., Rovers M., Riley R.D., Simmonds M., Stewart G., Tierney J.F., PRISMA-IPD Development Group (2015). Preferred reporting items for systematic review and meta-analyses of individual participant data: The PRISMA-IPD Statement. JAMA.

[18] Tang W., Li M., Lu X.-H., Liu H.W., Wang T. (2014). Phospholipids profiling and outcome of peritoneal dialysis patients. Biomarkers.

[19] Dunn W.B., Summers A., Brown M., Goodacre R., Lambie M., Johnson T., Wilkie M., Davies S., Topley N., Brenchley P. (2012). Proof-of-principle study to detect metabolic changes in peritoneal dialysis effluent in patients who develop encapsulating peritoneal sclerosis. Nephrol. Dial. Transplant..

[20] Guleria A., Bajpai N.K., Rawat A., Khetrapal C.L., Prasad N., Kumar D. (2014). Metabolite characterization in peritoneal dialysis effluent using high-resolution (1) H and (1) H-(13) C NMR Spectroscopy. Magn. Reson. Chem..

[21] Asano M., Ishii T., Hirayama A., Mizuno M., Suzuki Y., Sakata F., Akiyama S.I., Maruyama S., Soga T., Kinashi H. (2019). Differences in peritoneal solute transport rates in peritoneal dialysis. Clin. Exp. Nephrol..

[22] Csaicsich D., Lichtenauer A.M., Vychytil A., Kasper D.C., Herzog R., Aufricht C., Kratochwill K. (2015). Feasibility of metabolomics analysis of dialysate effluents from patients undergoing peritoneal equilibration testing. Perit. Dial. Int..

[23] Kratochwill K., Boehm M., Herzog R., Gruber K., Lichtenauer A.M., Kuster L., Csaicsich D., Gleiss A., Alper S.L., Aufricht C. (2016). Addition of alanyl-glutamine to dialysis fluid restores peritoneal cellular stress responses—A first-in-man trial. PLoS ONE.

[24] Wiesenhofer F.M., Herzog R., Boehm M., Wagner A., Unterwurzacher M., Kasper D.C., Alper S.L., Vychytil A., Aufricht C., Kratochwill K. (2018). Targeted metabolomic profiling of peritoneal dialysis effluents shows anti-oxidative capacity of alanyl-glutamine. Front. Physiol..

[25] Rainville P.D., Theodoridis G., Plumb R.S., Wilson I.D. (2014). Advances in liquid chromatography coupled to mass spectrometry for metabolic phenotyping. TrAC Trends Anal. Chem..

[26] Theodoridis G., Gika H.G., Wilson I.D. (2008). LC-MS-based methodology for global metabolite profiling in Metabonomics/Metabolomics. TrAC Trends Anal. Chem..

[27] Theodoridis G.A., Gika H.G., Want E.J., Wilson I.D. (2012). Liquid chromatography–mass spectrometry based global metabolite profiling: A review. Anal. Chim. Acta..

[28] Begou O., Gika H.G., Wilson I.D., Theodoridis G. (2017). Hyphenated MS-based targeted approaches in metabolomics. Analyst.

[29] Kalim S., Rhee E.P. (2017). An overview of renal metabolomics. Kidney Int..

[30] Vanholder R., Boelaert J., Glorieux G., Eloot S. (2015). New methods and technologies for measuring uremic toxins and quantifying dialysis adequacy. Semin. Dial..

[31] Ji-Choi J.Y., Yoon Y.J., Choi H.J., Park S.H., Kim C.D., Kim I.S., Kwon T.H., Do J.Y., Kim S.H., Ryu D.H. (2011). Dialysis modality-dependent changes in serum metabolites: Accumulation of inosine and hypoxanthine in patients on haemodialysis. Nephrol. Dial. Transplant..

[32] Goek O.N., Prehn C., Sekula P., Römisch-Margl W., Döring A., Gieger C., Heier M., Koenig W., Wang-Sattler R., Illig T. (2013). Metabolites associate with kidney function decline and incident chronic kidney disease in the general population. Nephrol. Dial. Transplant..

[33] Lopes Barreto D., Struijk D.G., Patel V., Preedy V. (2016). Peritoneal Effluent Biomarker Discovery in Peritoneal Dialysis: The Omics Era. Biomarkers in Kidney Disease: Methods, Discoveries and Applications.

[34] Davies S.J. (2000). Peritoneal Solute Transport—We know it is important, but what is It?. Nephrol. Dial. Transplant..

[35] Mäkinen V.P., Tynkkynen T., Soininen P., Forsblom C., Peltola T., Kangas A.J., Groop P.H., Ala-Korpela M. (2012). Sphingomyelin is associated with kidney disease in type 1 diabetes (The FinnDiane Study). Metabolomics.

[36] Pavoine C., Pecker F. (2009). Sphingomyelinases: Their regulationand roles in cardiovascular pathophysiology. Cardiovasc. Res..

[37] Hla T., Dannenberg A.J. (2012). Sphingolipid signaling in metabolic disorders. Cell Metab..

[38] Vychytil A., Herzog R., Probst P., Ribitsch W., Lhotta K., Machold-Fabrizii V., Wiesholzer M., Kaufmann M., Salmhofer H., Windpessl M. (2018). A randomized controlled trial of alanyl-glutamine supplementation in peritoneal dialysis fluid to assess impact on biomarkers of peritoneal health. Kidney Int..

[39] Cruzat V., Macedo Rogero M., Noel Keane K., Curi R., Newsholme P. (2018). Glutamine: Metabolism and immune function, supplementation and clinical translation. Nutrients.

